# Impact of frailty on postoperative delirium in ICU patients aged 65 and older: a systematic review

**DOI:** 10.1136/bmjopen-2025-108249

**Published:** 2026-01-22

**Authors:** Denise Schindele, John McDonough, Tilmann Müller-Wolff

**Affiliations:** 1 Institute of Nursing Science and Practice, Paracelsus Medical University, Salzburg, Austria; 2 Regionale Kliniken Holding RKH GmbH, Ludwigsburg, Germany; 3 University of North Florida Brooks College of Health, Jacksonville, Florida, USA

**Keywords:** Adult intensive & critical care, Delirium, Frail Elderly, Frailty, Intensive Care Units, Nursing Care

## Abstract

**Objectives:**

The objective was to assess whether frailty is associated with an increased risk of postoperative delirium (POD) in intensive care unit (ICU) patients aged 65 years and older.

**Design:**

A systematic review was conducted in accordance with Preferred Reporting Items for Systematic Review and Meta-Analysis guidelines. MEDLINE (via PubMed) and the Cochrane Library were searched for studies published between August 2014 and January 2025, assessing frailty with validated instruments and reporting POD during ICU stay. While the search strategy was not limited to a specific study design, only observational studies met the inclusion criteria. Study quality was appraised using the Newcastle-Ottawa Scale (NOS). Due to methodological heterogeneity, results were synthesised narratively.

**Setting:**

This review targeted the intensive care setting specifically, including studies conducted in hospital-based ICUs in various countries.

**Results:**

Of 655 records, five studies (n=3045) met inclusion criteria. Frailty prevalence ranged from 10% to 34.9%. Tools used included the Fried Frailty Scale, modified Frailty Index (mFI), FRAIL Scale (Fatigue, Resistance, Ambulation, Illnesses, and Loss of weight), Comprehensive Assessment of Frailty and Edmonton Frailty Scale. Frail patients had higher POD incidence and experienced more complications such as acute kidney injury, prolonged mechanical ventilation and reoperation. NOS scores ranged from 5 to 7, indicating moderate quality.

**Conclusion:**

Frailty appears to be associated with an increased risk of POD in ICU patients aged 65 and older. Given the limited number and heterogeneity of studies, further research is needed to validate this relationship and to inform targeted prevention strategies in critical care.

**Trial registration number:**

https://doi.org/10.17605/OSF.IO/7TWQ8

**STRENGTHS AND LIMITATIONS OF THIS STUDY**

- This systematic review adhered to Preferred Reporting Items for Systematic Review and Meta-Analysis guidelines and was prospectively registered, ensuring transparency and methodological rigour.
- A comprehensive and structured search strategy was developed, including both Medical Subject Headings terms and free-text keywords.
- Only observational studies using validated frailty instruments were included, enhancing the methodological consistency of frailty assessment.
- Heterogeneity in the tools used to assess frailty and diagnose postoperative delirium may have introduced classification bias and limited comparability.
- The database search was restricted to MEDLINE and the Cochrane Library, potentially omitting relevant studies indexed elsewhere.

## Introduction

 Surgical interventions are common among older adults and frequently require intensive care support. In Germany alone, over 6 million patients aged 65–80 underwent surgery in 2021, and more than 400 000 required intensive care treatment.^1^ This growing demand reflects demographic shifts and increasing surgical complexity in ageing populations.^2^

There is a long history of confusion being reported in postoperative patients.^3^ Older patients are at heightened risk of adverse outcomes in the postoperative period.^4^ While advanced age itself is a known risk factor, the presence of multimorbidity, functional decline and frailty significantly contribute to perioperative vulnerability.^5^ Frailty, in particular, represents a multidimensional geriatric syndrome characterised by a progressive decline in physiological reserves, leading to impaired homeostasis and increased susceptibility to stressors.^6^

The pathophysiological burden of frailty, when combined with surgical trauma, has been linked to a higher incidence of severe complications, including postoperative delirium (POD).^57^,^9^ POD is a serious and potentially preventable complication in perioperative and critical care.^10^ It is defined as an acute neuropsychiatric syndrome characterised by fluctuating disturbances in attention, awareness and cognition, often accompanied by disorganised thinking and altered consciousness. POD is associated with adverse outcomes, including prolonged hospitalisation, increased morbidity and mortality, and long-term cognitive decline.^11^ Prevalence estimates in intensive care settings vary widely, ranging from 20% to 80% depending on patient population and diagnostic methods.^12^ The aetiology of POD is complex and multifactorial. Several risk factors are specific to the intensive care context, including sedation, sleep disruption, immobility and iatrogenic complications.

Symptoms may be overlooked or misattributed to other neuropsychiatric disorders or medication effects, contributing to underdiagnosis.^12^ One of the predisposing factors is increased age,^12^ and with the increased age, frailty is a common factor.^6^

Existing systematic reviews and meta-analyses have examined the association between frailty and delirium in hospitalised older adults.^9 10 13 14^ A recent meta-analysis by Gracie *et al* (2021) demonstrated that frailty was associated with more than twice the odds of developing POD (OR 2.14, 95% CI 1.43 to 3.19).

However, these reviews predominantly included general hospital populations or mixed surgical cohorts and did not differentiate postoperative intensive care unit (ICU) patients from other settings. As a result, the applicability of their findings to critically ill postoperative adults remains limited.

The ICU differs substantially from general wards, with factors such as mechanical ventilation, sedative exposure, haemodynamic instability and acute physiological stressors playing a key role in delirium development.^12^ Despite these unique risks, no systematic review has focused specifically on the relationship between frailty and POD in postoperative ICU patients aged ≥65 years. Existing evidence therefore lacks a targeted synthesis for this distinct high-risk subgroup. Addressing this gap is essential to determine whether frailty consistently predicts POD under ICU-specific conditions and to support the development of tailored preventive strategies in critical care settings.

## Background

Frailty is a multidimensional geriatric syndrome that extends beyond chronological age and affects multiple physiological systems.^5 6^ It is characterised by diminished strength, endurance and physiological function, leading to increased vulnerability to stressors such as surgery and critical illness. Rather than reflecting a single organ dysfunction, frailty indicates a global decline in physiological reserve.^6^

Despite its clinical relevance, frailty remains difficult to assess in acute care contexts. In ICU settings, no universally accepted standard exists, and a wide variety of instruments are used.^15 16^

Many of the available frailty instruments are derived from the Fried frailty phenotype^6^ and the Clinical Frailty Scale (CFS).^17^ These tools vary in their complexity and clinical feasibility, which contributes to heterogeneity in frailty identification and risk stratification.^615^,^17^

Given the variability in frailty assessment across ICU settings, a clearer understanding of how different instruments perform in postoperative critical care may support more consistent risk stratification and guide the selection of feasible tools for routine clinical practice.

## Aim

The aim of this systematic review is to assess whether frailty is associated with an increased risk of POD in ICU patients aged 65 years and older.

The review addresses the question: Among postoperative ICU patients aged ≥65 years, is frailty associated with an increased risk of developing POD compared with non-frail patients?

## Design and methods

### patient and public involvement

Patients and the public were not involved in the design, conduct, reporting or dissemination plans of this systematic review.

### Research ethics approval

This study is a systematic review of previously published literature. No primary data were collected and no human participants were directly involved. Therefore, ethical approval was not required.

### Design

A systematic literature search following the Preferred Reporting Items for Systematic Review and Meta-Analysis (PRISMA) guidelines (online supplemental A)^18^ was conducted to identify relevant studies addressing the research question.

The protocol for the systematic review was duly registered in Open Science Framework (OSF) (registration: https://doi.org/10.17605/OSF.IO/7TWQ8). While the search strategy was not limited to a specific study design, only observational studies met the inclusion criteria.

Due to methodological differences across the included studies, particularly in the definitions and assessment of frailty and POD, we applied a narrative synthesis to summarise the findings.

### Search strategy

The literature search was conducted in MEDLINE (via PubMed) and the Cochrane Library. These databases were selected because they provide the most comprehensive indexing of clinical, perioperative and critical care literature, including the majority of studies relevant to frailty and delirium. Given the highly specific focus on postoperative ICU patients aged ≥65 years, the evidence base is primarily concentrated in these core biomedical databases. To enhance completeness, the reference lists of all included studies were also screened; however, no additional eligible studies were identified. The initial search took place between August and September 2024 and was updated in January 2025. We acknowledge that the restricted database selection may have limited the scope of retrieved studies.

The search strategy was systematically developed, incorporating Medical Subject Headings terms and free-text keywords. Key terms included ‘frailty’, ‘postoperative delirium’, ‘intensive care unit’ and ‘aged’. The search strings were customised for each database and combined using Boolean operators to ensure a comprehensive yet targeted retrieval of studies. A detailed description and the complete search strings of the search strategy are provided in the search protocol (online supplemental B). All search results were imported into a reference management software (EndNote V.21.5), and duplicates were removed prior to screening. In addition, the reference lists of all included studies were systematically screened to identify further eligible publications; however, no additional studies meeting the predefined inclusion criteria were identified.

### Inclusion and exclusion criteria

The eligibility criteria were refined as follows:

1. Observational studies (prospective or retrospective cohort studies) published between 2014 and 2025. (Rationale: Only observational studies are suitable for assessing associations relevant to the research question.)
2. Patients aged ≥65 years, assessed with a validated frailty instrument (eg, Fried Frailty Scale, Edmonton Frailty Scale, FRAIL Scale Fatigue, Resistance, Ambulation, Illnesses, and Loss of weight), modified Frailty Index (mFI), CFS).
3. Studies investigating postoperative adult patients treated in an ICU, irrespective of surgical discipline.
4. Studies reporting POD occurring during the ICU stay, assessed using validated tools (eg, confusion assessment method (CAM), CAM-ICU) or documented clinical diagnosis. (No restrictions were applied regarding delirium assessment method.)

Exclusion criteria:

1. Studies not involving surgical patients (eg, medical ICU admissions).
2. Studies including patients younger than 65 years.
3. Interventional trials targeting frailty improvement and validation-only studies not reporting POD outcomes.
4. Studies focusing exclusively on long-term outcomes (eg, 6-month cognitive follow-up) without postoperative ICU delirium data.
5. Studies in which patients did not receive ICU treatment during the postoperative period.
6. Studies that did not report delirium as an outcome.

Additional exclusions:

Abstracts, conference abstracts, qualitative studies, case reports, secondary analyses and studies not published in English or German.

Systematic reviews and meta-analyses were excluded from the primary analysis but used for contextualisation of the evidence base.

### Study selection

The study selection process followed a structured approach to ensure methodological rigour. Initially, duplicate records and grey literature were removed. Subsequently, two independent reviewers (DS and TMW) screened the titles and abstracts for relevance, applying the predefined inclusion and exclusion criteria. Any discrepancies between the reviewers were resolved through discussion.

Following this, a full-text screening was conducted using the same independent review process. Studies that did not meet the eligibility criteria were excluded, with reasons for exclusion systematically documented. The final selection of studies was verified for consistency, ensuring that all included studies aligned with the review’s objectives.

### Quality appraisal

Quality assessment of the included observational studies was conducted using the Newcastle-Ottawa Scale (NOS), which evaluates methodological quality across three domains: selection, comparability and outcome assessment.^19^ Two reviewers independently applied the NOS criteria, with disagreements resolved through discussion. No modifications to the original tool were made.

### Data extraction

Data extraction was performed independently by two reviewers using a standardised extraction sheet. Extracted variables included study characteristics (author, year, country, sample size, setting), patient demographics, frailty assessment tools, POD diagnosis methods and ICU outcomes.

### Synthesis

A synthesis without meta-analysis approach was used to summarise the results of the included studies. Data were categorised based on frailty assessment tools, delirium diagnosis methods and reported ICU outcomes to facilitate structured comparison.

## Results

**Figure 1:**
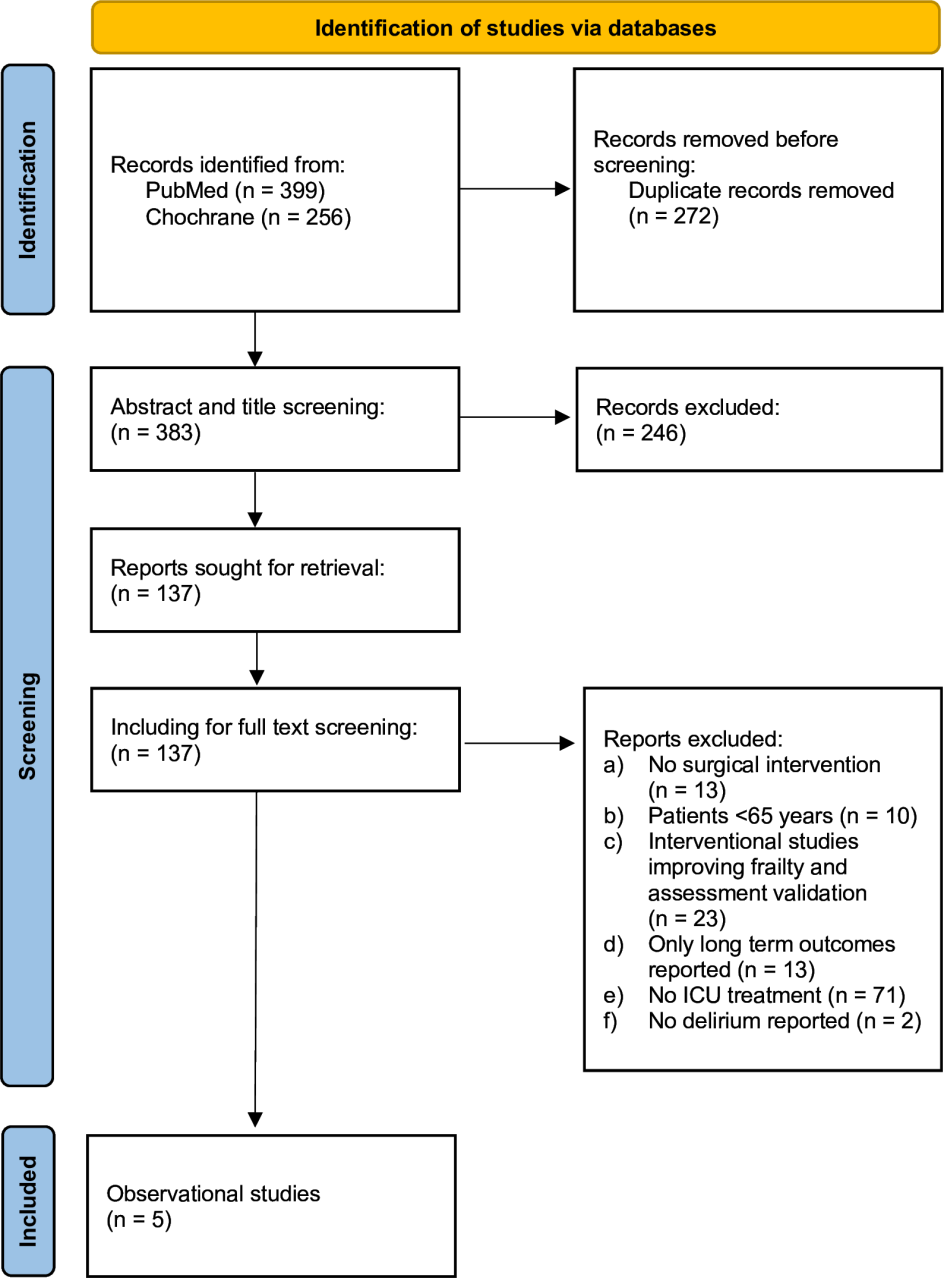
Search results, PRISMA flow diagram. ICU, intensive care unit; PRISMA, Preferred Reporting Items for Systematic Review and Meta-Analysis.

Out of 655 identified search results, five studies were included. The results are shown in the PRISMA flowchart (figure 1).^18^

### Quality appraisal

The methodological quality of the included studies was moderate, with NOS scores ranging from 5 to 7. Several potential sources of bias were identified. The representativeness of the exposed cohort varied, as some studies focused on specific patient groups, such as those undergoing cardiac surgery, which may limit the generalisability of findings to broader ICU populations. While most studies accounted for major confounders, variability in frailty assessment methods and delirium diagnostic criteria introduced heterogeneity, potentially affecting comparability. Differences in delirium diagnosis were evident, with some studies using standardised tools such as the CAM or CAM-ICU, whereas others relied on clinical judgement, leading to potential inconsistencies in outcome classification. Additionally, the follow-up periods varied, with some studies lacking long-term delirium outcome data, which may have influenced the ability to assess POD recurrence and its prolonged effects. Despite these methodological limitations, the overall study quality suggests that the findings are robust. However, caution is warranted when interpreting the results due to the heterogeneity in study designs and outcome assessment methods.

No study was excluded from the systematic review on the basis of its quality (online supplemental C). The table 1 reports the evaluation of selection, comparability and outcome domains. Higher scores indicate higher methodological quality. Follow-up periods and adequacy of outcome assessment are reflected in the corresponding NOS domains.

**Table 1:** Data extraction and study characteristics

Author	n	ICU discipline	Frailty score	Delirium score	Primary outcomes	ICU complications	Follow-up
Bäck *et al* ^21^	604	Elective and non-elective cardiac surgery	CAF (frailty prevalence 25%)	Not reported	Mortality	AKI, need for reoperation, delirium, MV	30 days after surgery
López Cuenca *et al*^24^	132	Interdisciplinary ICU	FRAIL Scale (frailty prevalence 34.9%)	Not reported	Mortality	AKI, bleeding, delirium	1 and 6 months after ICU discharge
Nomura *et al*^23^	133	Cardiac surgery	Fried Frailty Scale (frailty prevalence 33,1%)	CAM, CAM-ICU	Delirium	Mortality, hospital LOS	1 and 12 months after surgery
Lal *et al*^22^	96	Cardiac surgery	EFS (frailty prevalence 10%)	Not reported	Hospital LOS	AKI, re-sternotomy, delirium	12 months after ICU discharge
Cheng *et al*^20^	2080	Cardiac surgery	mFI (frailty prevalence 29.5%)	CAM-ICU	Delirium	MV, mortality, hospital LOS	Not reported

AKI, acute kidney injury; CAF, comprehensive assessment of frailty; CAM, confusion assessment method; CAM-ICU, confusion assessment method for ICU; EFS, Edmonton Frailty Scale; FRAIL, Fatigue, Resistance, Ambulation, Illnesses, and Loss of weight; ICU, intensive care unit; LOS, length of stay; mFI, modified Frailty Index; MV, controlled mechanical ventilation; n, population.

### Study selection and characteristics

Table 1 presents the main characteristics and the extracted data of the included studies, including sample size, surgical discipline, frailty and delirium assessment methods, primary outcomes, follow-up periods and reported ICU-related complications. A comprehensive version of this table, including additional variables such as study design, age criteria and country, is provided in the supplementary material (online supplemental D). In table 1 frailty prevalence refers exclusively to patients classified as frail according to the respective assessment tool. Prefrail individuals were not included in these proportions. A detailed breakdown of frailty categories is provided in the section ‘Frailty Assessment Tools and Prevalence’.

The five included studies showed similar demographic patterns, with patients typically aged 73–78 years and predominantly male (68–84%). Most cohorts consisted of individuals undergoing major cardiac surgery.^20^,^23^

### Frailty assessment tools and prevalence

The included studies employed a variety of validated tools to assess frailty, reflecting diverse approaches tailored to the multifaceted nature of the condition.^20^,^24^ In the study by Bäck *et al*^21^ 604 patients were assessed using the comprehensive assessment of frailty, a multifaceted tool that integrates physical performance tests (eg, gait speed and grip strength) with clinical evaluations of comorbidities and nutritional status. This instrument integrates objective metrics and clinician judgement to classify patients as non-frail, prefrail or frail. In their study, 25% of the patients were classified as frail and 30% as prefrail.^21^

Nomura *et al*^23^ employed the Fried Frailty Scale in 133 patients, a tool that assesses frailty across five domains: unintentional weight loss, weakness, exhaustion, slowness and low physical activity. Patients were categorised as non-frail, prefrail or frail based on their cumulative scores. The Fried Frailty Scale is predicated on a phenotypic model of frailty, emphasising physical capabilities. In this study, 33.1% of the patients were classified as frail and 55.6% as prefrail.^23^

The FRAIL Scale, used by López Cuenca *et al*^24^ in 132 patients, uses five simple criteria: fatigue, resistance, ambulation, illness and weight loss. This instrument categorises individuals as robust (0 points), prefrail (1–2 points) or frail (3–5 points), thereby providing a straightforward and expeditious assessment method. The study reported that 34.9% of the ICU patients were classified as frail and 16.7% as prefrail.^24^

Lal *et al*^22^ applied the Edmonton Frail Scale (EFS) in 96 patients, which evaluates frailty across multiple domains, including cognitive function, physical performance, medication use and social support. The EFS offers a comprehensive yet efficient assessment suitable for non-specialists, who can use it to classify patients as non-frail, prefrail or frail based on their scores. In the present cohort, 10% of the patients were categorised as frail and 23% as prefrail.^22^

In the only retrospective study by Cheng *et al*^20^ 2080 patients were screened by the adopted mFI, a tool based on a deficit accumulation model. This index integrates 11 health-related factors, including comorbidities and functional status, to calculate a cumulative score. Patients were grouped into two categories: non-frail (mFI 0–2) or frail (mFI ≥3). This approach underscores a more comprehensive, multidimensional perspective. Among the 2080 participants, 29.5% were identified as frail.^20^

### Incidence of POD

The included studies examined the incidence of POD in relation to frailty, with varying associations.^20^,^24^

In the study by Bäck *et al*^21^ POD was identified through clinical assessment. No validated instrument was used. The incidence of POD was found to be 7% in patients with frailty compared with 3% in patients without frailty. The analysis revealed a relationship between frailty and an elevated risk of POD, with an OR of 5.0 (95% CI 1.6 to 16.0; p=0.006).^21^

Nomura *et al*^23^ employed the CAM and the adapted version for intensive care units, the CAM-ICU, to diagnose POD, reporting an incidence of 48% in frail and prefrail patients, compared with 13% in non-frail patients. The adjusted OR for POD in frail patients was 6.31 (95% CI 1.18 to 33.74; p=0.03).^23^

López Cuenca *et al*^24^ identified delirium through routine clinical documentation by the ICU team, without the use of a structured delirium screening tool such as CAM or CAM-ICU, and without a formal validation procedure. The incidence of POD was documented to be 11% in patients categorised as frail and 12% in those designated as non-frail. No relationship was found between frailty and delirium (OR 0.93, 95% CI 0.30 to 2.89, p=0.57).

In the study by Lal *et al,*^22^ the CAM-ICU was used to diagnose delirium. The incidence was 10% in frail patients, 14% in vulnerable patients and 5% in non-frail patients. While frail patients exhibited a higher incidence of delirium, the association between frailty and delirium was not statistically significant (OR: 1.25, 95% CI 0.92 to 1.71, p=0.161).^22^

Cheng *et al*^20^ employed the CAM-ICU, augmented by nursing notes, to diagnose delirium. The prevalence of delirium was 29.2% in patients with frailty and 16.4% in patients without frailty. Frailty was associated with POD, with an adjusted OR of 1.61 (95% CI 1.23 to 2.10).^20^

Overall, the findings indicate a consistent trend of increased POD among frail patients. However, variations in delirium diagnostic methods, study designs and patient populations may contribute to differences in reported POD incidence. Table 2 summarises the ORs and CIs for POD in frail patients compared with non-frail individuals.

**Table 2:** ORs for POD in frail patients

Study	Delirium prevalence in frail patients	OR for POD (95% CI)	P value
Bäck *et al*^21^	7%	5.0 (1.6 to 16.0)	0.006
Nomura *et al*^23^	48%	6.31 (1.18 to 33.74)	0.03
López Cuenca *et al*^24^	11%	0.93 (0.30 to 2.89)	0.57
Lal *et al*^22^	10%	1.25 (0.92 to 1.71)	0.161
Cheng *et al*^20^	29.2%	1.61 (1.23 to 2.10)	<0.01

POD, postoperative delirium.

### ICU-related outcomes

Furthermore, the five studies examined several postoperative complications in addition to POD.^20^,^24^ Frail patients demonstrated higher rates of acute kidney injury (AKI),^21 22 24^ prolonged mechanical ventilation^20 21^ and reoperation due to bleeding or other causes compared with non-frail patients.^21 22 24^ Frail patients also have a higher mortality rate and a prolonged hospitalisation compared with non-frail patients.^20^,^24^

## Discussion

This review suggests that frailty may be associated with an increased risk of POD in ICU patients aged 65 years and older. Reported POD incidence ranged from 7% to 48%, with frail individuals consistently showing higher rates. In addition, frailty was linked to complications such as AKI, prolonged mechanical ventilation and reoperation.

Substantial methodological heterogeneity was identified due to differences in population, frailty measures, delirium assessments and follow-up periods across the studies. Frailty was assessed using various instruments, ranging from phenotype-based tools (eg, Fried Frailty Scale) to multidimensional models (eg, Edmonton Frailty Scale) and delirium was diagnosed using different approaches, including validated instruments (CAM, CAM-ICU) and unstructured clinical assessments. These inconsistencies likely contributed to variation in outcomes and complicated direct comparisons. Across the included studies, frailty was operationalised using diverse tools, including the Fried Frailty Scale, FRAIL Scale, Edmonton Frailty Scale, mFI and the CAF, reflecting the absence of a uniform frailty construct in ICU research. This variability affects comparability across studies and highlights the broader challenge of establishing consensus on frailty measurement in critical care populations.^25 26^

The findings are broadly consistent with previous research showing that frailty contributes to adverse ICU outcomes, including delirium, prolonged length of stay and mortality.^527^,^31^

Although the association between frailty and POD is well supported in general hospital populations, most previous studies did not specifically investigate ICU cohorts, highlighting the need for research focused on critically ill patients.^7 9 13 14 32^

This review identified only five eligible ICU studies.^20^,^24^

Four were conducted in cardiac surgery populations,^20^,^23^ which limits the generalisability of the findings to broader surgical populations. Only one study included an interdisciplinary ICU cohort,^24^ indicating that evidence for non-cardiac surgical patients remains sparse.

Cardiac surgery-related factors (eg, extracorporeal circulation) may influence delirium risk differently from other specialties,^33^ further restricting external validity.

Recent evidence from a multicentre study by Dólera Moreno *et al*^34^ indicates that frailty is also associated with adverse outcomes in predominantly medical ICU populations. This suggests that the prognostic relevance of frailty may differ between surgical and medical ICU settings due to their distinct clinical pathways.^34^

Recent findings from Molina Lobo *et al*^35^ further indicate that frailty and even prefrailty may be prognostically relevant across mixed ICU populations of all ages. Although not specific to postoperative patients, these findings reinforce that frailty is not confined to older adults and support population-specific analyses.^35^ The meta-analysis conducted by Gracie *et al*^36^ reported a more than twofold increased odds of POD in frail patients. Their pooled analysis of nine studies demonstrated that frail patients had more than twice the odds of developing POD compared with non-frail patients (OR=2.14, 95% CI 1.43 to 3.19). Importantly, this analysis also highlighted the relatively low heterogeneity (I²=5.5), suggesting robust findings despite variations in frailty and delirium assessment tools.^36^ These results are broadly consistent with the findings of the present review, although important methodological differences between both analyses must be considered. While the meta-analysis by Gracie *et al*^36^ included nine studies, several of these were not eligible for our review, which applied stricter criteria requiring postoperative ICU admission, validated frailty assessment and delirium occurring during the ICU stay. These differences explain the smaller number of included studies and reflect the narrower clinical focus of the present review. Studies in mixed-age ICU populations^37 38^ also reported higher delirium rates among frail patients, although population differences limit comparability with postoperative cohorts.

In interpreting these findings, several methodological limitations should be considered. First, the number of eligible studies was small (n=5), limiting generalisability and reducing the robustness of conclusions. Second, the predominance of cardiac surgery cohorts constrains applicability to other surgical ICU populations. Third, substantial heterogeneity in frailty instruments, delirium assessment methods and follow-up periods precluded quantitative synthesis.

Moreover, none of the included studies applied the CFS, despite its widespread use in intensive care medicine as a validated and clinically established frailty measure.^25^ This absence limits comparability with the broader ICU frailty literature and may have contributed to variation in frailty classification across studies.

In addition, only two of the included studies evaluated delirium as a predefined primary endpoint. In contrast, the studies by Bäck *et al*^21^ and Lal *et al*^22^ assessed delirium as a secondary outcome using systematic clinical or tool-based assessments, whereas López Cuenca *et al*^24^ documented delirium solely as a routine clinical complication without a structured screening procedure. The absence of predefined delirium endpoints in three studies reduces statistical power and increases the risk of outcome misclassification, thereby limiting the strength of conclusions regarding the association between frailty and POD. Fourth, methodological quality was moderate (NOS scores 5–7), with limited adjustment for important confounders such as baseline cognitive impairment, anticholinergic burden, premedication and comorbidity burden. Finally, differences in study design and reporting may have introduced residual confounding. Together, these limitations indicate that the evidence base remains preliminary and should be interpreted with caution.

Another important source of potential bias relates to baseline cognitive impairment. Dementia and preoperative cognitive decline are strong predictors of delirium and may confound the association between frailty and POD if not adequately assessed or adjusted for. Several frailty instruments, particularly multidimensional measures, partially capture cognitive components, which may increase their predictive value for delirium but also blur the distinction between frailty and pre-existing cognitive deficits.^39 40^ Future studies should therefore include standardised preoperative cognitive assessments to improve comparability and reduce misclassification.

In addition, pharmacological and clinical factors such as anticholinergic burden, premedication (eg, benzodiazepines or sedatives) and comorbidities may influence delirium risk and could act as confounders, as previously demonstrated in studies identifying anticholinergic load and sedative exposure as established delirium risk factors^41^ and comorbidity burden as a contributor to delirium vulnerability.^29^ Most included studies provided limited reporting or adjustment for these variables, which may have contributed to residual confounding. Standardised documentation of medication profiles and comorbidity burden would strengthen the interpretability of future research.

Overall, this review suggests that frailty may represent a clinically relevant marker for increased POD risk in postoperative ICU patients aged ≥65 years. Recognising frailty as a potential vulnerability factor may support closer monitoring and targeted supportive measures, although evidence for frailty-specific interventions remains limited. Future studies should include broader surgical populations, apply standardised frailty and delirium assessments, and evaluate whether tailored interventions can reduce POD risk in frail ICU patients.

## Conclusion

Frailty is a potential risk factor of POD in ICU patients aged 65 years and older. Across the included studies, frail individuals tended to exhibit higher rates of POD and postoperative complications; however, the strength of this association varied between studies.

In clinical practice, frailty may serve as one component within a broader, multifactorial delirium risk assessment, helping to identify older ICU patients who may benefit from enhanced monitoring and non-pharmacological preventive strategies. However, the current evidence does not allow firm recommendations regarding specific interventions.

Future studies should include more diverse surgical populations, apply standardised and validated frailty and delirium assessment tools and evaluate whether targeted perioperative interventions can mitigate POD risk in frail patients. Improved methodological consistency will be essential to strengthen the evidence base and support the development of robust clinical recommendations.

### Implications and recommendations for practice

- Early identification: Frailty screening may be integrated into preoperative and ICU workflows, where feasible, to help identify older patients who could benefit from closer monitoring regarding delirium risk.
- Multidisciplinary collaboration: A multidisciplinary approach involving geriatricians, intensivists and general and advanced nursing staff may support more comprehensive care for frail ICU patients, acknowledging the complexity of their clinical needs.
- Standardisation: The use of standardised and validated frailty and delirium assessment tools could improve comparability across studies and strengthen the evidence base for future clinical decision-making.
- Targeted prevention strategies: Enhanced delirium monitoring, multimodal pain management and supportive interventions to maintain cognitive and physical activity may contribute to reducing POD risk in frail older adults, although further research is required to determine their effectiveness in ICU settings.

## Supplementary material

10.1136/bmjopen-2025-108249 online supplemental file 1

10.1136/bmjopen-2025-108249 online supplemental file 2

10.1136/bmjopen-2025-108249 online supplemental file 3

10.1136/bmjopen-2025-108249 online supplemental file 4

## Data Availability

All data relevant to the study are included in the article or uploaded as supplementary information.
