# Supplementary material for: Impact of frailty on postoperative delirium in ICU patients aged 65 and older: a systematic review
Source: BMJ Open. 2026 Jan 22;16(1):e108249. doi: 10.1136/bmjopen-2025-108249 (PMC12829360; doi:10.1136/bmjopen-2025-108249)
Supplement: online supplemental file 2 [file bmjopen-16-1-s002.pdf]

Supplement B: Search protocol for the systematic literature search as part of a systematic review

General information on the development of the search strategy 2
Research Question 2
Including and excluding criteria 2
1 Defining the search principles 2
2 Definition of search terms 3
3 Definition of databases to be searched 3
4 Identification of key words 3
5 Identification of index terms 4
6 Developing the search string 5
8 Conducted the research and screening 6
9 Documentation of the research 7
  9.1 Medline via Pubmed 7
  9.2 Cochrane Library 7

Source:

Hirt, J., Nordhausen, T. (2022). Rechercheprotokoll für eine systematische Literaturrecherche. In: Nordhausen, T., Hirt, J. RefHunter. Systematische Literaturrecherche. https://refhunter.org/research_support/rechercheprotokoll/ [17.05.2023]

Version 4.0
30. Juni 2022

General information on the development of the search strategy

Name oft the researcher: Denise Schindele, Tilmann Müller-Wolff
Date or period of development: 01.08.2024 – 30.09.2024, updated January 2025

Research Question

Does the geriatric syndrome of frailty constitute a risk factor for postoperative delirium in patients over the age of 65 years who are undergoing intensive care?

Including and excluding criteria

Domain	Including	Excluding
**Domain 1: Study design**	observational studies, systematic reviews, meta-analyses, randomized controlled trials (RCT) and clinically controlled trials (CCT)	abstracts, case reports, conference proceedings, qualitative reports, secondary and subanalyses
**Domain 2: Publication year**	2014 - 2024	
**Domain 3: Population**	Patients ≥65 years and older with frailty	Pat. <65 years, pat. without frailty degree, no frailty assessed, no valid assessment instrument used
**Domain 4: Outcome**	Postoperative delirium	no surgical intervention, no survey of complications during hospital and ICU stay, no ICU stay, long-term outcomes
**Domain 5: language**	german and english	Other language

Justification for some including and excluding criteria:

☒ Study design: prevalence of complications was the outcome of interest and the highest possible level of evidence should be presented.

Population: the evidence regarding the relationship between frailty and postoperative delirium should be presented. Therefore, the survey of frailty is mandatory for the analysis

Outcome: the outcome postoperative delirium/postoperative complications during the ICU stay should be considered

1 Defining the search principles

☒ Mixed form (partially sensitive or specific)

Goal: Find as many relevant hits as possible with an optimized effort ratio

2 Definition of search terms

Search terms	Term
**Search terms 1**	Pat. >65 years
**Search terms 2**	Frailty
**Search terms 3**	Postoperative delirium
**Search terms 4**	Intensive care unit

3 Definition of databases to be searched

Database	Term
	**justification**
**Database 1**	MEDLINE via PubMed
	important database for medical literature and can be used as a primary database, broad spectrum in the health sector, broad spectrum of research designs
**Database 2**	Cochrane Library
	a comprehensive database of systematic reviews and meta-analyses, including scoping reviews, broad spectrum of intervention studies, reviews, broad spectrum healthcare, evidence-based decision making

4 Identification of key words

Search terms	Key words
**Search terms 1: Pat. >65 years**	AGED<br>ELDERLY
**Search terms 2: Frailty**	FRAIL<br>FRAILTY SYNDROM
**Search terms 3: postoperative delirium**	POSTOP. OUTCOME<br>DELIRIUM
**Search terms 4: Intensive care unit**	ICU<br>CRITICAL CARE UNIT

5 Identification of index terms

Identification of index terms for the key words defined in the previous step for each search component and database.

Key words	Search terms	Index terms: Medline via Pubmed	Index terms: Cochrane Library		
AGED<br>ELDERLY	**Search terms 1:**<br>**Pat. >65 years**	AGED	AGED		
FRAIL<br>FRAILTY SYNDROM	**Search terms 2: Frailty**	FRAIL ELDERLY<br>FRAILTY	FRAIL ELDERLY<br>FRAILTY		
POSTOP. OUTCOME<br>DELIRIUM	**Search terms 3:**<br>**postoperative delirium**	POSTOPERATIVE COMPLICATIONS<br>DELIRIUM	POSTOPERATIVE COMPLICATIONS<br>DELIRIUM		
ICU<br>CRITICAL CARE UNIT	**Search terms 4:**<br>**Intensive care unit**	INTENSIV CARE UNITS	INTENSIV CARE UNITS		

Justification for the index terms:

☒ Medline via Pubmed: MeshTerms were identified as index terms addition to the key words. MeshTerms ensure a targeted search.
Cochrane Library: see above

6 Developing the search string

Search term	Search string: **Pubmed**	Search string: **Cochrane**		
**Search term 1: Pat. >65 years**	AGED OR<br><br>ELDERLY	AGED OR<br><br>ELDERLY		
	AND	AND	AND	
**Search term 2: Frailty**	FRAIL ELDERLY OR<br><br>FRAILTY	FRAIL ELDERLY OR<br><br>FRAILTY		
	AND	AND	AND	
**Search term 3: postoperative delirium**	POSTOPERATIVE COMPLICATIONS OR<br><br>CRITICAL CARE OUTCOMES<br><br>DELIRIUM	POSTOPERATIVE COMPLICATIONS OR<br><br>CRITICAL CARE OUTCOMES<br><br>DELIRIUM		
	OR/AND	OR/AND	OR	
**Search terms 4: Intensive care unit**	INTENSIV CARE UNITS	INTENSIV CARE UNITS		

.

8 Conducted the research and screening

The predefined inclusion criteria function as search filters:

RCT, CCT, observational studies, meta-analysen, systematic reviews
Patients aged 65 and over with frailty
Postoperative delirium, within the postoperative phase and the hospital stay/ICU stay
German and english
2014 - 2024

Prior to the title and abstract screening, any duplicates were removed, and grey literature was excluded

Exclusion criteria for abstract/titel screening:

- The title and abstract must not indicate any signs of frailty
- Frailty was not assessed using a validated assessment tool
- The text is in a language other than that specified
- The study excluded patients who had undergone surgical procedures
- Absence of an intensive care unit (ICU) stay

Exclusion criteria for full text analysis:

- The inclusion of intervention studies aimed at improving frailty is also permitted
- Studies concerned with the validation of assessment instruments
- The analysis was limited to long-term outcomes
- No outcomes of relevance to the intensive care unit
- The exclusion criterion was the absence of an ICU stay
- The study did not include any patients who had undergone surgical procedures

9 Documentation of the research

The documentation of the database-specific search strings should include details of the date of the search and the number of search hits.

9.1 Medline via Pubmed

Search date: 15.08.2024, Updated 10.01.2025

#	Input	Number of hits
1	FRAILTY AND POSTOPERATIVE DELIRIUM AND INTENSIVE CARE UNIT<br><br>(("FRAILTY"[MESH TERMS] OR "FRAILTY"[ALL FIELDS] OR "FRAILTIES"[ALL FIELDS]) AND ("EMERGENCE DELIRIUM"[MESH TERMS] OR ("EMERGENCE"[ALL FIELDS] AND "DELIRIUM"[ALL FIELDS]) OR "EMERGENCE DELIRIUM"[ALL FIELDS] OR ("POSTOPERATIVE"[ALL FIELDS] AND "DELIRIUM"[ALL FIELDS]) OR "POSTOPERATIVE DELIRIUM"[ALL FIELDS]) AND ("INTENSIVE CARE UNITS"[MESH TERMS] OR ("INTENSIVE"[ALL FIELDS] AND "CARE"[ALL FIELDS] AND "UNITS"[ALL FIELDS]) OR "INTENSIVE CARE UNITS"[ALL FIELDS] OR ("INTENSIVE"[ALL FIELDS] AND "CARE"[ALL FIELDS] AND "UNIT"[ALL FIELDS]) OR "INTENSIVE CARE UNIT"[ALL FIELDS])) AND (OBSERVATIONALSTUDY[FILTER])	11
2	(AGED OR AGED, 80 AND OVER OR ELDERLY) AND (FRAIL ELDERLY OR FRAILTY) AND (CRITICAL CARE OUTCOMES OR INTENSIV CARE UNITS)<br><br>(("AGED"[MESH TERMS] OR "AGED"[ALL FIELDS] OR ("AGED"[MESH TERMS] OR "AGED"[ALL FIELDS] OR "ELDERLY"[ALL FIELDS] OR "ELDERLIES"[ALL FIELDS] OR "ELDERLY S"[ALL FIELDS] OR "ELDERLYS"[ALL FIELDS])) AND ("FRAIL ELDERLY"[MESH TERMS] OR ("FRAIL"[ALL FIELDS] AND "ELDERLY"[ALL FIELDS]) OR "FRAIL ELDERLY"[ALL FIELDS] OR ("FRAILTY"[MESH TERMS] OR "FRAILTY"[ALL FIELDS] OR "FRAILTIES"[ALL FIELDS])) AND ("CRITICAL CARE OUTCOMES"[MESH TERMS] OR ("CRITICAL"[ALL FIELDS] AND "CARE"[ALL FIELDS] AND "OUTCOMES"[ALL FIELDS]) OR "CRITICAL CARE OUTCOMES"[ALL FIELDS] OR ("INTENSIV"[ALL FIELDS] AND "CARE"[ALL FIELDS] AND ("UNIT S"[ALL FIELDS] OR "UNITS"[ALL FIELDS])))) AND (OBSERVATIONALSTUDY[FILTER])	142
3	(AGED OR ELDERLY) AND (FRAIL ELDERLY OR FRAILTY) AND (POSTOPERATIVE OUTCOMES OR INTENSIV CARE UNITS)<br><br>(((("AGED"[MESH TERMS] OR "AGED"[ALL FIELDS] OR (("AGED"[MESH TERMS] OR "AGED"[ALL FIELDS]) AND "80"[ALL FIELDS])) AND "OVER"[ALL FIELDS]) OR ("AGED"[MESH TERMS] OR "AGED"[ALL FIELDS] OR "ELDERLY"[ALL FIELDS] OR "ELDERLIES"[ALL FIELDS] OR "ELDERLY S"[ALL FIELDS] OR "ELDERLYS"[ALL FIELDS])) AND ("FRAIL ELDERLY"[MESH TERMS] OR ("FRAIL"[ALL FIELDS] AND "ELDERLY"[ALL FIELDS]) OR "FRAIL ELDERLY"[ALL FIELDS] OR ("FRAILTY"[MESH TERMS] OR "FRAILTY"[ALL FIELDS] OR "FRAILTIES"[ALL FIELDS])) AND ((("POSTOPERATIVE PERIOD"[MESH TERMS] OR ("POSTOPERATIVE"[ALL FIELDS] AND "PERIOD"[ALL FIELDS]) OR "POSTOPERATIVE PERIOD"[ALL FIELDS] OR "POSTOP"[ALL FIELDS] OR "POSTOPERATIVE"[ALL FIELDS] OR "POSTOPERATIVELY"[ALL FIELDS] OR "POSTOPERATIVES"[ALL FIELDS]) AND ("OUTCOME"[ALL FIELDS] OR "OUTCOMES"[ALL FIELDS])) OR ("INTENSIV"[ALL FIELDS] AND "CARE"[ALL FIELDS] AND ("UNIT S"[ALL FIELDS] OR "UNITS"[ALL FIELDS])))) AND ((CONTROLLEDCLINICALTRIAL[FILTER] OR META-ANALYSIS[FILTER] OR OBSERVATIONALSTUDY[FILTER] OR RANDOMIZEDCONTROLLEDTRIAL[FILTER] OR SYSTEMATICREVIEW[FILTER]) AND (ENGLISH[FILTER] OR GERMAN[FILTER]) AND (AGED[FILTER]) AND (2010:2023[PDAT]))	246
	TOTAL	399

9.2 Cochrane Library

Search date: 15.08.2024, Updated 10.01.2025

#	Input	Number of hits
1	FRAILTY AND POSTOPERATIVE DELIRIUM AND INTENSIVE CARE UNIT	16
2	(AGED OR ELDERLY) AND (FRAIL ELDERLY OR FRAILTY) AND (CRITICAL CARE OUTCOMES OR INTENSIV CARE UNITS)	103
4	(AGED OR ELDERLY) AND (FRAIL ELDERLY OR FRAILTY) AND (POSTOPERATIVE OUTCOMES OR INTENSIV CARE UNITS)	137
	TOTAL	256
