# Supplementary material for: Impact of frailty on postoperative delirium in ICU patients aged 65 and older: a systematic review
Source: BMJ Open. 2026 Jan 22;16(1):e108249. doi: 10.1136/bmjopen-2025-108249 (PMC12829360; doi:10.1136/bmjopen-2025-108249)
Supplement: online supplemental file 3 [file bmjopen-16-1-s003.docx]

Supplement C

Critical appraisal of included observational studies using the Newcastle–Ottawa Scale

| Author + Year | Selection of study participants | | | | Comparability | Outcome | | | Points |
| --- | --- | --- | --- | --- | --- | --- | --- | --- | --- |
|  | Representativeness of the exposed cohort | Selection of the non-exposed cohort | Determination of exposure | Demonstration that outcome of interest was not present at start of study | Comparability of cohorts on the basis of the design or analysis controlled for confounders | Assessment of outcome | Was follow-up long enough for outcomes to occur | Adequacy of follow-up of cohorts |  |
| Bäck et al.  2019 |  | * | * | * | * | * | * |  | 6 |
| López Cuenca et al. 2019 |  | * | * | * | * | * | * | * | 7 |
| Nomura et al. 2019 |  | * | * | * | * | * | * |  | 6 |
| Lal et al.  2020 |  | * | * | * | * | * | * |  | 6 |
| Cheng et al. 2024 |  | * | * | * |  | * |  |  | 5 |
