# Supplementary material for: Impact of frailty on postoperative delirium in ICU patients aged 65 and older: a systematic review
Source: BMJ Open. 2026 Jan 22;16(1):e108249. doi: 10.1136/bmjopen-2025-108249 (PMC12829360; doi:10.1136/bmjopen-2025-108249)
Supplement: online supplemental file 4 [file bmjopen-16-1-s004.docx]

Supplement D

Data extraction and study characteristics – extended version

| Author + year | Study design | Country | n | ICU discipline | Age | Frailty score | Delirium score | Primary outcomes | ICU complications | Follow-up |
| --- | --- | --- | --- | --- | --- | --- | --- | --- | --- | --- |
| Bäck et al.  2019 | prospective observational | Denmark | 604 | elective and non-elective cardiac surgery | ≥65 years | CAF (frailty prevalence 25%) | not reported | mortality | AKI, need for reoperation, delirium, MV | 30 days after surgery |
| López Cuenca et al. 2019 | prospective observational | Spain | 132 | interdisciplinary ICU | ≥65 years | FRAIL Scale (frailty prevalence 34,9%) | not reported | mortality | AKI, bleeding, delirium | one and six months after ICU discharge |
| Nomura et al. 2019 | prospective observational | USA | 133 | cardiac surgery | ≥65 years | Fried Frailty Scale (frailty prevalence 30%) | CAM, CAM-ICU | delirium | mortality, hospital LOS | one and 12 months after surgery |
| Lal et al.  2020 | prospective observational | New Zealand | 96 | cardiac surgery | ≥65 years | EFS (frailty prevalence 10%) | not reported | hospital LOS | AKI, re-sternotomy, delirium | 12 months after ICU discharge |
| Cheng et al.  2024 | retrospective observational | USA | 2080 | cardiac surgery | ≥65 years | mFI (frailty prevalence 29,5%) | CAM-ICU | delirium | MV, mortality, hospital LOS | not reported |

Abbreviations: AKI: acute kidney injury; CAM: confusion assessment instrument; CAM-ICU: confusion assessment instrument for ICU; CAF: Comprehensive Assessment of Frailty; EFS: Edmonton Frailty Scale; LOS: length of stay; mFI: modified frail index; MV: controlled mechanical ventilation; ICU: intensive care unit; n: Population
