# Supplementary material for: Impact of frailty on postoperative delirium in ICU patients aged 65 and older: a systematic review
Source: BMJ Open. 2026 Jan 22;16(1):e108249. doi: 10.1136/bmjopen-2025-108249 (PMC12829360; doi:10.1136/bmjopen-2025-108249)
Supplement: Supplementary file 5 [file bmjopen-2025-108249.reviewer_comments.pdf]

PEER REVIEW HISTORY

BMJ Open publishes all reviews undertaken for accepted manuscripts. Reviewers are asked to complete a checklist review form and are provided with free text boxes to elaborate on their assessment. These free text comments are reproduced below.

ARTICLE DETAILS

Title (Provisional)

Impact of Frailty on Postoperative Delirium in ICU Patients Aged 65 and Older: A Systematic Review

Authors

Schindele, Denise; McDonough, John; Müller-Wolff, Tilmann

VERSION 1 - REVIEW

Reviewer 1

Name Fuest, Kristina

Affiliation Technische Universität München, Anaesthesiology & Imtensive Care Medicine

Date 05-Sep-2025

COI None

Reviewer comment

Thank you for the opportunity to review this manuscript. The topic of 'frailty and postoperative delirium in intensive care patients' is clinically relevant, and the authors have tried to align the work with PRISMA standards. The research question is interesting, but I see some key points that should be addressed before publication.

Main point of criticism

• Justification for the review ('added value') is missing: Several systematic reviews and meta-analyses have already investigated the association between frailty and delirium. The specific insights gained from this review compared to the existing literature remain unclear. Although reference is made to the ICU context, it must be clarified why a further systematic review is necessary and what contribution it makes beyond the existing evidence. This includes the fact that although the introduction covers the two topics of frailty and POD, the existing evidence (from other SRs or MAs) is not presented. What is the state of the literature on this?

Further points of criticism

Formal and linguistic aspects

• Numerous spelling errors (e.g., Chochrane, electice, cardiosurgery, diver's approaches). These give the impression of sloppy work and should be carefully corrected. What does "subacute cardiosurgery" mean?

• Language mixing and inconsistencies (German/English (“Neuseeland”), upper/lower case) should be eliminated.

• Abbreviations are introduced several times and used inconsistently in some cases.

Structure and content

• The separation between the Introduction and the Background is unclear, as the content overlaps significantly. Streamlining and a clear structure would significantly enhance the readability and accessibility of the manuscript, making it easier for readers to navigate and understand the content.

• The description of the inclusion and exclusion criteria is confusing and should be presented more clearly, stringently, and with reference to the research question.

• The PICO question (population, intervention/exposure, comparison, outcome) that the review is intended to answer must be formulated explicitly and precisely.

• The tables are poorly prepared: table legends are missing or incomplete, and there are typos (‘included’ instead of ‘including’ as well as the mixed languages etc. see above). In addition, key information, such as follow-up periods, is missing from the results tables. The discussion of the results is generally insufficient: it primarily focuses on cardiac surgery studies, and the generalisability to other surgical populations remains unclear. Although a bias assessment is provided (Newcastle-Ottawa Scale), it is dealt with too briefly and is not sufficiently integrated into the discussion. What is the author’s resume?

Content points

• Why was the CFS (Clinical Frailty Scale) not considered, even though it is widely used in intensive care medicine? This should be addressed in the discussion.

• The difference to the meta-analysis by Gracie et al. (2021) (9 studies vs. five here) is not explained convincingly.

• When addressing issues in the category of age, frailty, and delirium, the bias caused by dementia must be clearly highlighted. The preoperative cognitive state is essential; here, it may improve the informative value of some frailty scales. In addition, factors such as anticholinergic burden, pre-medication, and comorbidities play a role and should be reflected in the review.

Summary

The manuscript addresses a relevant topic, but currently suffers from formal deficiencies, poor table formatting, and an overly superficial discussion. Above all, however, the scientific rationale for this review (compared to existing reviews and meta-analyses) needs to be clarified significantly.

Recommendation: Major revision

**Reviewer**	**2**
**Name**	**Gordo, Federico**

Affiliation **Hospital Universitario del Henares, Department of Intensive Care**

Date **27-Sep-2025**

COI **None**

The authors present a systematic review, without meta-analysis, aimed at evaluating whether frailty is associated with an increased risk of postoperative delirium (POD) in patients aged 65 years or older admitted to intensive care units (ICUs).

The systematic review adheres to PRISMA guidelines and was prospectively registered, which strengthens the methodological rigor. The search was conducted for studies published between August 2014 and January 2025 in MEDLINE (PubMed) and the Cochrane Library. However, it is not specified whether iterative searches of references were performed, which could have enhanced comprehensiveness. Only observational studies using validated instruments to assess frailty and reporting on the incidence of POD during ICU stay were included. Methodological quality was evaluated using the Newcastle–Ottawa Scale (NOS), and results were synthesized narratively due to methodological heterogeneity.

Of the 655 records initially identified, five studies (n = 3,045) met the inclusion criteria. The prevalence of frailty ranged between 25% and 34.9%. Various frailty assessment tools were used: the Fried Frailty Index, the Modified Frailty Index (mFI), the FRAIL Scale, the Comprehensive Assessment of Frailty, and the Edmonton Frailty Scale. Frail patients showed a higher incidence of POD as well as complications such as acute kidney injury, prolonged mechanical ventilation, and reoperation. NOS scores ranged from 5 to 7, indicating moderate methodological quality.

The authors conclude that frailty appears to be associated with a higher risk of POD in older ICU patients. Nevertheless, given the scarcity and heterogeneity of available studies, further research is required to confirm this association and to inform tailored preventive strategies in critical care.

The topic addressed is highly relevant, given population aging and the substantial burden of complications in older ICU patients. Establishing a clear link between frailty and POD could have a meaningful impact on clinical practice.

The main limitation of this review is the small number of included studies—only five—which limits generalizability and reduces the robustness of the conclusions. The discussion could be expanded to better highlight the clinical implications, particularly regarding prevention and management of delirium in frail patients.

For instance, Dolera et al. (10.1016/j.medin.2024.06.003) recently published a multicenter study within the review's search window, examining the effect and incidence of frailty across all ages. They found that frailty was associated with mortality, particularly in patients with medical (non-surgical) ICU admissions. This raises the question of whether frailty behaves differently in surgical versus medical ICU populations—a point worth discussing.

Similarly, Molina et al. (DOI: 10.1016/j.medin.2025.502144) evaluated frailty incidence in a mixed ICU population of all ages (median age 66 years). Their findings show that frailty can

develop at any age, not only in older adults, and that both frailty and even pre-frailty may be associated with prognosis. Addressing these recent findings would strengthen the discussion and contextualize the current review within the broader literature.

VERSION 1 - AUTHOR RESPONSE

Response Letter to Reviewer #2

Thank you for the opportunity to review this manuscript. The topic of ‘frailty and postoperative delirium in intensive care patients’ is clinically relevant, and the authors have tried to align the work with PRISMA standards. The research question is interesting, but I see some key points that should be addressed before publication.

Main point of criticism:

Comment 1: Justification for the review (‘added value’) is missing: Several systematic reviews and meta-analyses have already investigated the association between frailty and delirium. The specific insights gained from this review compared to the existing literature remain unclear. Although reference is made to the ICU context, it must be clarified why a further systematic review is necessary and what contribution it makes beyond the existing evidence. This includes the fact that although the introduction covers the two topics of frailty and POD, the existing evidence (from other SRs or MAs) is not presented. What is the state of the literature on this?

We thank the reviewer for this insightful comment. We agree that a clear justification of the added value of this review is essential.

Specifically, we now state that previous reviews (e.g., Persico et al., 2018; Fu et al., 2022; Cechinel et al., 2022; Zhang et al., 2021; Gracie et al., 2021) focused on general or mixed hospital populations and did not analyse postoperative ICU cohorts separately. We also clarify that the ICU environment differs substantially from general wards, and that no prior review has examined the association between frailty and POD specifically in postoperative ICU patients aged ≥65 years.

To avoid redundancy, we revised and shortened the Background.

Formal and linguistic aspects:

Comment 2: Numerous spelling errors (e.g., Chochrane, electice, cardiosurgery, diver’s approaches). These give the impression of sloppy work and should be carefully corrected. What does “subacute cardiosurgery” mean?

Thank you for pointing this out. We carefully reviewed the entire manuscript and corrected all spelling errors, including the examples mentioned (“Cochrane”, “elective”, “cardiac surgery”, “diverse approaches”). We also clarified the terminology “subacute cardiosurgery” and replaced it with the more widely used expression “elective and urgent cardiac surgery” to improve accuracy and readability.

In addition, we conducted a full language check of the manuscript and corrected several further minor spelling and terminology inconsistencies.

Comment 3: Language mixing and inconsistencies (German/English (“Neuseeland”), upper/lower case) should be eliminated.

Thank you for this helpful comment. We carefully reviewed the manuscript for language mixing and eliminated all remaining German terms, including “Neuseeland,” which has been corrected to “New Zealand.” We also harmonised upper/lower-case usage and standardised terminology (e.g., “LOS,” “Delirium Score,” “cardiac surgery”). In addition, we corrected several minor linguistic inconsistencies across tables and text to ensure a fully consistent and professional use of English throughout the manuscript.

Comment 4: Abbreviations are introduced several times and used inconsistently in some cases.

Thank you for your comment. We reviewed all abbreviations throughout the manuscript, ensured that each abbreviation is introduced only once, and standardised their use across the entire text and tables. Inconsistencies (e.g., LOS/LoS, ICU terminology, Delirium Score) have been corrected.

Structure and content

Comment 5: The separation between the Introduction and the Background is unclear, as the content overlaps significantly. Streamlining and a clear structure would significantly enhance the readability and accessibility of the manuscript, making it easier for readers to navigate and understand the content.

Thank you for this comment. We streamlined the Introduction and Background to eliminate overlaps and improve readability. The Introduction now presents the full context, existing evidence, and the knowledge gap, while the Background has been substantially shortened to avoid repetition and to focus only on essential methodological aspects.

Comment 6: The description of the inclusion and exclusion criteria is confusing and should be presented more clearly, stringently, and with reference to the research question.

Thank you for this helpful comment. We redefined the inclusion and exclusion criteria to ensure clear alignment with the research question and to enhance readability. The section was restructured for greater stringency, redundancies were removed, and eligibility criteria are now presented in a consistent, logically ordered format. This revision clearly differentiates inclusion from exclusion criteria and explicitly links each criterion to the target population, setting, and outcome of interest.

Comment 7: The PICO question (population, intervention/exposure, comparison, outcome) that the review is intended to answer must be formulated explicitly and precisely.

Thank you for this important comment. We have now added an explicit and precise PICO question to clearly define the population, exposure, comparison group, and outcome of interest. This addition

clarifies the focus of the review and ensures that the research question is fully aligned with established methodological standards for systematic reviews. The revised PICO question has been inserted after the Aim section.

Comment 8: The tables are poorly prepared: table legends are missing or incomplete, and there are typos ('included' instead of 'including' as well as the mixed languages etc. see above). In addition, key information, such as follow-up periods, is missing from the results tables. The discussion of the results is generally insufficient: it primarily focuses on cardiac surgery studies, and the generalisability to other surgical populations remains unclear. Although a bias assessment is provided (Newcastle-Ottawa Scale), it is dealt with too briefly and is not sufficiently integrated into the discussion. What is the author's resume?

Thank you very much for this comprehensive and constructive feedback. We fully agree with your observations and have implemented extensive revisions to address each of the issues raised:

Tables: We thoroughly revised all tables to improve readability and accuracy. Table legends were added or completed, remaining language inconsistencies and typographical errors were corrected, and a new *Follow-up* column was added to Table 2 based on verified information extracted directly from the original studies. This enhances transparency and completeness of the reported study characteristics.

Language and consistency: All remaining language inconsistencies (including mixed German/English terminology, upper/lower case issues, and typographical errors) were corrected throughout the manuscript to ensure a professional and coherent presentation.

Discussion: The Discussion section was substantially restructured to improve clarity, depth, and alignment with the data. We expanded the interpretation of results, clarified the predominance of cardiac surgery cohorts and the resulting limitations in generalisability, and highlighted the scarcity of evidence from non-cardiac surgical ICU populations. In addition, the findings were more explicitly situated within the broader literature.

Integration of bias assessment: The results of the Newcastle–Ottawa Scale (NOS) quality appraisal were integrated into the Discussion. We now comment explicitly on methodological limitations, sources of heterogeneity, and the moderate overall quality of included studies, ensuring that the strength of the evidence is appropriately contextualised.

Clear summary ("author's résumé"): Thank you for this valuable comment. We have substantially revised the Conclusion to provide a clearer and more balanced overall résumé of the review findings. The revised section now (1) summarises the main results, (2) explicitly acknowledges limitations regarding generalisability, methodological heterogeneity and risk of bias, and (3) outlines cautious clinical implications and priorities for future research.

Content points

Comment 9: Why was the CFS (Clinical Frailty Scale) not considered, even though it is widely used in intensive care medicine? This should be addressed in the discussion.

Thank you for this helpful comment. We have added a clarification to the Discussion explaining why the Clinical Frailty Scale (CFS) was not included in the synthesis.

None of the eligible studies applied the CFS as their frailty assessment tool, which prevented its consideration in the review. We now explicitly acknowledge the widespread use and clinical relevance of the CFS in intensive care medicine and highlight the need for future ICU studies to incorporate standardized frailty measures, including the CFS, to enhance comparability and clinical applicability.

Comment 10: The difference to the meta-analysis by Gracie et al. (2021) (9 studies vs. five here) is not explained convincingly.

Thank you for this comment. We have clarified in the Discussion why the present review includes five studies, whereas the meta-analysis by Gracie et al. (2021) included nine. Gracie et al. applied broader inclusion criteria, synthesising mixed surgical populations and studies conducted on general wards or combined ward/ICU settings, without restricting delirium assessment to the postoperative ICU stay. In contrast, our review focused exclusively on postoperative ICU patients aged ≥65 years with frailty assessed using validated instruments and delirium occurring specifically during the ICU admission. Several studies included by Gracie et al. therefore did not meet the ICU-specific eligibility criteria and were not eligible for inclusion. This explanation has now been added to the manuscript directly after the Gracie et al. paragraph.

Comment 11: When addressing issues in the category of age, frailty, and delirium, the bias caused by dementia must be clearly highlighted. The preoperative cognitive state is essential; here, it may improve the informative value of some frailty scales. In addition, factors such as anticholinergic burden, pre-medication, and comorbidities play a role and should be reflected in the review.

Thank you for this important observation. We have expanded the Discussion to explicitly address key sources of potential bias related to age, frailty, and delirium. In the revised manuscript, we now highlight the confounding impact of baseline cognitive impairment and dementia, referencing established evidence that these factors are among the strongest predictors of delirium. We also discuss how cognitive components embedded in some frailty instruments may influence the association between frailty and POD.

Furthermore, we added a statement on additional confounders—including anticholinergic burden, pre-medication (e.g., benzodiazepines or sedatives), and comorbidities—and clarified that these factors were insufficiently reported or adjusted for in the included studies. Their potential contribution to residual confounding is now acknowledged, along with recommendations for standardized assessment in future research.

Summary:

The manuscript addresses a relevant topic, but currently suffers from formal deficiencies, poor table formatting, and an overly superficial discussion. Above all, however, the scientific rationale for this review (compared to existing reviews and meta-analyses) needs to be clarified significantly.

Recommendation: Major revision

Response Letter to Reviewer #2

The authors present a systematic review, without meta-analysis, aimed at evaluating whether frailty is associated with an increased risk of postoperative delirium (POD) in patients aged 65 years or older admitted to intensive care units (ICUs).

Comment 1: The systematic review adheres to PRISMA guidelines and was prospectively registered, which strengthens the methodological rigor. The search was conducted for studies published between August 2014 and January 2025 in MEDLINE (PubMed) and the Cochrane Library. However, it is not specified whether iterative searches of references were performed, which could have enhanced comprehensiveness. Only observational studies using validated instruments to assess frailty and reporting on the incidence of POD during ICU stay were included. Methodological quality was evaluated using the Newcastle–Ottawa Scale (NOS), and results were synthesized narratively due to methodological heterogeneity.

Thank you for this helpful comment. We have clarified the search process in the revised manuscript. In addition to the database searches, we systematically screened the reference lists of all included studies to identify further eligible publications. No additional studies meeting the predefined inclusion criteria were found. This information has now been added to the Search Strategy section to enhance transparency and comprehensiveness.

Of the 655 records initially identified, five studies (n = 3,045) met the inclusion criteria. The prevalence of frailty ranged between 25% and 34.9%. Various frailty assessment tools were used: the Fried Frailty Index, the Modified Frailty Index (mFI), the FRAIL Scale, the Comprehensive Assessment of Frailty, and the Edmonton Frailty Scale. Frail patients showed a higher incidence of POD as well as complications such as acute kidney injury, prolonged mechanical ventilation, and reoperation. NOS scores ranged from 5 to 7, indicating moderate methodological quality.

The authors conclude that frailty appears to be associated with a higher risk of POD in older ICU patients. Nevertheless, given the scarcity and heterogeneity of available studies, further research is required to confirm this association and to inform tailored preventive strategies in critical care.

The topic addressed is highly relevant, given population aging and the substantial burden of complications in older ICU patients. Establishing a clear link between frailty and POD could have a meaningful impact on clinical practice.

Comment 2: The main limitation of this review is the small number of included studies—only five—which limits generalizability and reduces the robustness of the conclusions. The discussion could be expanded to better highlight the clinical implications, particularly regarding prevention and management of delirium in frail patients.

Thank you for this comment. We have expanded the Discussion to more clearly emphasise that the small number of included studies represents a major limitation, restricting generalisability and reducing the robustness of the conclusions. In addition, we have elaborated on the clinical

implications of our findings, particularly with regard to the prevention and management of delirium in frail ICU patients. These additions now provide a more balanced interpretation and clearer guidance for clinical context, while remaining consistent with the limitations of the available evidence.

Comment 3: For instance, Dolera et al. (10.1016/j.medin.2024.06.003) recently published a multicenter study within the review's search window, examining the effect and incidence of frailty across all ages. They found that frailty was associated with mortality, particularly in patients with medical (non-surgical) ICU admissions. This raises the question of whether frailty behaves differently in surgical versus medical ICU populations—a point worth discussing.

Thank you for this comment. We have expanded the Discussion to more clearly emphasise that the small number of included studies represents a major limitation, restricting generalisability and reducing the robustness of the conclusions. In addition, we have elaborated on the clinical implications of our findings, particularly with regard to the prevention and management of delirium in frail ICU patients. These additions now provide a more balanced interpretation and clearer guidance for clinical context, while remaining consistent with the limitations of the available evidence.

Comment 4: Similarly, Molina et al. (DOI: 10.1016/j.medin.2025.502144) evaluated frailty incidence in a mixed ICU population of all ages (median age 66 years). Their findings show that frailty can develop at any age, not only in older adults, and that both frailty and even pre-frailty may be associated with prognosis. Addressing these recent findings would strengthen the discussion and contextualize the current review within the broader literature.

Thank you for this helpful suggestion. We have added a brief discussion of the recent study by Molina et al. (2025), which examined frailty in a mixed ICU population across all ages. Although their cohort did not focus on postoperative patients, their findings reinforce that frailty and even pre-frailty may be prognostically relevant beyond older adults. We now highlight that these broader observations support the need for population-specific analyses and help to contextualize the focus of our review on surgical ICU patients aged ≥65 years.

VERSION 2 - REVIEW

**Reviewer**	**1**
**Name**	**Fuest, Kristina**
**Affiliation**	**Technische Universität München, Anaesthesiology & Imtensive Care Medicine**
**Date**	**05-Dec-2025**
**COI**	

Reviewer Comment to

bmjopen-2025-108249.R1 „Impact of Frailty on Postoperative Delirium in ICU Patients Aged 65 and Older: A Systematic Review“

Thank you for the extensive revision of the manuscript, which has clearly advanced the work and improved its overall readability. Nevertheless, the manuscript still requires substantial further refinement before it can be considered for publication.

- First, the section on the exclusion criteria remains incomplete, as exclusion criterion (f) is missing

- The PRISMA Flowchart might be graphically enhanced and formatted (equally aligned boxes)

- There is a redundancy that should be addressed: "The five included studies exhibited consistent demographic trends, characterizing a profile of older patients, predominantly male, undergoing major cardiac surgery. The mean age ranged from 73 to 78 years, and the gender distribution exhibited a predominance of male patients, with proportions ranging from 68% to 84%. The surgical procedures were predominantly high-risk cardiac procedures."

- Several issues persist in the tables. Beyond the previously mentioned example of cardiac/Cardiac, there are multiple inconsistencies in capitalization across table entries that need to be standardized.

- Additionally, the prevalence of frailty in each included study should be presented in Table 1, as this information is essential for a comprehensive descriptive overview. Similarly, the prevalence of delirium in each included study should be included in Table 2, given its central relevance to the research question.

- In the Discussion section, the added paragraph addressing the Clinical Frailty Scale (CFS) should be moved to the Limitations section

- A clarification is also needed regarding the description of the study by López Cuenca et al. The manuscript states: "López Cuenca et al. (2019) also assessed POD based on clinical diagnosis and validated file reviews…". It is unclear what is meant by clinical diagnosis and how validated file reviews were performed.

- In addition, the following sentence is grammatically incomplete and should be corrected: "Although the association between frailty and POD is well supported in general hospital populations and most previous studies did not specifically investigate ICU populations."

- An important limitation is still missing from the manuscript. While the authors do not explicitly state it, the overview table indicates that only two of the included studies evaluated delirium as a primary endpoint, and at least one large study (e.g., Bäck et al., with more than 600 patients) did not include delirium, even as a secondary endpoint. This issue should be explicitly addressed in the Limitations section, as it has direct implications for statistical power and the strength of the conclusions regarding the association between frailty and postoperative delirium.

Finally, several language and formatting issues remain throughout the manuscript. These include spelling errors, inconsistent capitalization in tables, inconsistent spacing in in-text

citations (e.g., sometimes including a space before the bracket, sometimes not), as well as double spaces and other typographical inconsistencies. A careful, comprehensive proofreading is again strongly recommended.

VERSION 2 - AUTHOR RESPONSE

Response Letter to Reviewer # 1

Thank you for the extensive revision of the manuscript, which has clearly advanced the work and improved its overall readability. Nevertheless, the manuscript still requires substantial further refinement before it can be considered for publication.
Thank you very much for your positive feedback regarding the substantial revisions already undertaken. We sincerely appreciate your acknowledgement that the manuscript has improved in clarity and overall readability. We also take note of your assessment that further refinement is required before the work can be considered for publication.

First, the section on the exclusion criteria remains incomplete, as exclusion criterion (f) is missing
Thank you for the helpful remark. The omission has been corrected, and exclusion criterion (f) has now been explicitly added to the Methods section.

- The PRISMA Flowchart might be graphically enhanced and formatted (equally aligned boxes)
Thank you for this helpful suggestion. The PRISMA flowchart has been graphically revised using the official PRISMA template to ensure a clearer and more consistent presentation. Should the visual appearance still not meet the journal’s preferences, we would be happy to work with the editorial team to further refine the formatting in line with the journal’s graphical standards.

- There is a redundancy that should be addressed: “The five included studies exhibited consistent demographic trends, characterizing a profile of older patients, predominantly male, undergoing major cardiac surgery. The mean age ranged from 73 to 78 years, and the gender distribution exhibited a predominance of male patients, with proportions ranging from 68% to 84%. The surgical procedures were predominantly high-risk cardiac procedures.”

Thank you for highlighting this redundancy. The sentences have been consolidated to remove repetition and now present the demographic characteristics in a concise and coherent form.
„The five included studies showed similar demographic patterns, with patients typically aged 73–78 years and predominantly male (68–84%). Most cohorts consisted of individuals undergoing major cardiac surgery“

- Several issues persist in the tables. Beyond the previously mentioned example of cardiac/Cardiac, there are multiple inconsistencies in capitalization across table entries that need to be standardized.

Thank you for pointing out the remaining inconsistencies in capitalization within the tables. We have carefully reviewed all table entries and standardized capitalization throughout to ensure consistent and correct formatting.

- Additionally, the prevalence of frailty in each included study should be presented in Table 1, as this information is essential for a comprehensive descriptive overview. Similarly, the prevalence of delirium in each included study should be included in Table 2, given its central relevance to the research question.

Thank you for this helpful comment. We have now added the delirium prevalence for each study to Table 2 as requested. In Table 1, the frailty prevalence has been integrated within the respective frailty assessment entries in accordance with the journal's formatting requirements regarding table width and column limitations.

- In the Discussion section, the added paragraph addressing the Clinical Frailty Scale (CFS) should be moved to the Limitations section

Thank you for pointing out the redundancy regarding the paragraph on the Clinical Frailty Scale (CFS). We have revised the manuscript accordingly. The CFS-related content has been relocated to the Limitations section, where it is now more appropriately contextualised. The corresponding paragraph in the Discussion has been rewritten to avoid overlap and now focuses solely on the broader heterogeneity of frailty and delirium assessment methods.

- A clarification is also needed regarding the description of the study by López Cuenca et al. The manuscript states: "López Cuenca et al. (2019) also assessed POD based on clinical diagnosis and validated file reviews…". It is unclear what is meant by clinical diagnosis and how validated file reviews were performed.

Thank you for noting the unclear description of delirium assessment in the study by López Cuenca et al. We have revised the manuscript accordingly. The text now clarifies that delirium in this study was identified through routine clinical documentation by the ICU team, without the use of a structured screening tool or a formal validation procedure.

- In addition, the following sentence is grammatically incomplete and should be corrected: "Although the association between frailty and POD is well supported in general hospital populations and most previous studies did not specifically investigate ICU populations."

The sentence has been revised to ensure clarity and completeness.

- An important limitation is still missing from the manuscript. While the authors do not explicitly state it, the overview table indicates that only two of the included studies evaluated delirium as a primary endpoint, and at least one large study (e.g., Bäck et al., with more than 600 patients) did not include delirium, even as a secondary endpoint. This issue should be explicitly addressed in the Limitations section, as it has direct implications for statistical power and the strength of the conclusions regarding the association between frailty and postoperative delirium.

Thank you for highlighting this important limitation. We have now revised the Limitations section to explicitly acknowledge that only two of the included studies evaluated delirium as a predefined primary endpoint, while the remaining studies assessed delirium either as a secondary outcome or, in the case of López Cuenca et al., based on routine clinical documentation. As noted, this variation reduces statistical power and may increase the risk of outcome misclassification, thereby limiting the strength of conclusions regarding the frailty–delirium association.

Finally, several language and formatting issues remain throughout the manuscript. These include spelling errors, inconsistent capitalization in tables, inconsistent spacing in in-text citations (e.g., sometimes including a space before the bracket, sometimes not), as well as double spaces and other typographical inconsistencies. A careful, comprehensive proofreading is again strongly recommended.

Thank you for drawing attention to the remaining language and formatting inconsistencies. We have carefully re-reviewed the entire manuscript and corrected all identified issues, including spelling errors, inconsistent capitalization in tables,

spacing irregularities in in-text citations, typographical inconsistencies, and double spaces.
